# Targeting Cancer Hallmarks Using Selected Food Bioactive Compounds: Potentials for Preventive and Therapeutic Strategies

**DOI:** 10.3390/foods13172687

**Published:** 2024-08-26

**Authors:** Wamidh H. Talib, Ilia Abed, Daniah Raad, Raghad K. Alomari, Ayah Jamal, Rand Jabbar, Eman Omar Amin Alhasan, Heba K. Alshaeri, Moudi M. Alasmari, Douglas Law

**Affiliations:** 1 Faculty of Allied Medical Sciences, Applied Science Private University, Amman 11931, Jordan; emi_spice27@yahoo.com; 2 Faculty of Health and Life Sciences, Inti International University, Nilai 71800, Negeri Sembilan, Malaysia; douglas.law@newinti.edu.my; 3 Department of Clinical Pharmacy and Therapeutics, Applied Science Private University, Amman 11931, Jordan; elyaazireeni@gmail.com (I.A.); danyabushnaq@gmail.com (D.R.); alomariraghad98@gmail.com (R.K.A.); aya.rahahleh@yahoo.com (A.J.); ranood.aljbouri@gmail.com (R.J.); 4 Department of Pharmacology, Faculty of Medicine, King Abdul-Aziz University, Rabigh 25724, Saudi Arabia; 5 College of Medicine, King Saud bin Abdulaziz University for Health Sciences (KSAU-HS), Jeddah 21423, Saudi Arabia; asmarim@ksau-hs.edu.sa; 6 King Abdullah International Medical Research Centre (KAIMRC), Jeddah 22233, Saudi Arabia

**Keywords:** cancer hallmarks, foods, anticancer, polyphenols, human health, spices, vegetables, bioactive compounds

## Abstract

Cancer continues to be a prominent issue in healthcare systems, resulting in approximately 9.9 million fatalities in 2020. It is the second most common cause of death after cardiovascular diseases. Although there are difficulties in treating cancer at both the genetic and phenotypic levels, many cancer patients seek supplementary and alternative medicines to cope with their illness, relieve symptoms, and reduce the side effects of cytotoxic drug therapy. Consequently, there is an increasing emphasis on studying natural products that have the potential to prevent or treat cancer. Cancer cells depend on multiple hallmarks to secure survival. These hallmarks include sustained proliferation, apoptosis inactivation, stimulation of angiogenesis, immune evasion, and altered metabolism. Several natural products from food were reported to target multiple cancer hallmarks and can be used as adjuvant interventions to augment conventional therapies. This review summarizes the main active ingredients in food that have anticancer activities with a comprehensive discussion of the mechanisms of action. Thymoquinone, allicin, resveratrol, parthenolide, Epigallocatechin gallate, and piperine are promising anticancer bioactive ingredients in food. Natural products discussed in this review provide a solid ground for researchers to provide effective anticancer functional food.

## 1. Introduction

In the last twenty years, there has been a significant rise in the occurrence of cancer, which refers to a disrupted series of biological processes that harm human body cells and cause them to multiply excessively [1]. According to data from 2020, the global number of deaths caused by cancer was approximately 10 million, while there were 19.3 million newly diagnosed cases of cancer [2]. Cancer, being the second leading cause of death globally, imposes a substantial economic burden on public healthcare systems [3]. Regional and national disparities in the occurrence, death rate, and burden of cancer measured in disability-adjusted life years (DALY) are substantial, especially when comparing developed and developing nations [2]. Cancer rates, deaths, and the impact on years of healthy life (DALYs) vary greatly due to multiple factors, such as ecological, environmental, demographic, cultural, and genetic influences [4].

A statistical study conducted between 2000 and 2017 analyzed cancer epidemiology in five Eastern Mediterranean countries, namely Jordan, Lebanon, Palestine, Syria, and Iraq, with a specific focus on total incidence rates. Lebanon exhibits the highest rates of occurrence compared to other countries, with a steady rise from 231 to 353. The average incidence rate in Syria is 112, and it has remained relatively stable. When comparing, it is evident that Palestine and Jordan exhibit strikingly similar outcomes, with average incidence rates of 143 and 137, respectively. Nevertheless, the rates in Iraq have consistently declined, decreasing from 126 per 100,000 in 2000 to 81 per 100,000 in 2017 [5,6].

Global cancer statistics consistently demonstrate that as much as 80% of the cancer incidence in high-income nations can be avoided. Moreover, it is estimated that around 50% of cancer cases can be prevented. Several key factors are essential for preventing cancer, including adhering to a nutritious diet, participating in regular physical activity, quitting smoking, and maintaining a healthy weight [7,8,9]. Therefore, the examination of the impact of dietary modifications, particularly on the commencement and progression of cancer, is a profoundly captivating subject [7]. Remarkably, dietary factors are responsible for precisely 5% of cancer cases. The increasing attention given to the correlation between food and cancer is the reason behind this phenomenon [10].

A recent study conducted in China investigated the relationship between the intake of fresh fruits and various causes of death. The study encompassed a grand total of 462,342 participants and documented 17,894 fatalities. The study found a correlation between consuming fresh fruit and a reduced risk of various causes of death, such as ischemic heart disease, stroke, other cardiovascular diseases, esophageal, stomach, and colon cancers, chronic obstructive pulmonary disease, other major chronic diseases, and all other causes of death [11]. Citrus fruits, with their high levels of flavanones and vitamin C, possess the capacity to efficiently inhibit a range of cancer types [12,13,14]. Moreover, there is a correlation between the consumption of vegetables and a reduced mortality rate from cancer in individuals who have previously received a cancer diagnosis [15]. Vegetables classified as cruciferous and green-yellow have the strongest association with a decreased risk of cancer compared to other types of vegetables [16]. The chemopreventive properties of isothiocyanates and carotenoids may be the main reason behind it [17].

Scientific research on saffron’s anti-tumor and chemopreventive properties has been growing. It has been found that saffron influences immune signals, apoptosis, the inhibition of DNA and RNA synthesis, and cellular proliferation. Moreover, it impedes the proliferation of cancer cells and exhibits antioxidant characteristics. Ginger, like saffron, exerts both direct and indirect effects on the viability of tumor cells while not inflicting substantial damage to the cells [18]. In addition, it improves the efficacy of anti-cancer medications and effectively acts as a complementary agent for different phytochemical components, such as garlic and turmeric [19].

This review aims to study bioactive compounds in food that have anticancer effects with a detailed discussion of the mechanisms of action. Additionally, it analyses different diets that include these foods in order to determine which cancer hallmark they can target and to elucidate the potential preventive mechanisms of these foods.

## 2. Methodology

The literature related to the topic of this review was collected through a careful search of the electronic databases. Web of Science, Scopus, PubMed, and Google Scholar were the databases used in this study. The following keywords were used during the search: “cancer hallmarks”, “Anticancer activity”, “Anticancer foods”, “Anticancer bioactive compounds”, “Mechanism of action”, “in vitro”, and “in vivo”.

## 3. The Hallmarks of Cancer

Cancers display significant disparities among various organs, including differences in genetic profiles, histopathology, systemic physiological effects, prognosis, and response to treatments. The intricate and varied characteristics of cancer, both in terms of genetics and observable traits, present a substantial obstacle for the field of cancer medicine. Extensive efforts have been made to uncover fundamental principles and mechanistic commonalities hidden within the genetic and phenotypic complexities of different types of cancer [20]. The hallmarks of cancer are a set of functional traits that describe the abilities human cells acquire as they transition from normal states to neoplastic growth states. The precise characteristics mentioned here are crucial in enabling cells to initiate the development of cancerous tumors [21]. The hallmarks of cancer serve as a fundamental framework for understanding the complexities of the disease and improving our understanding of its different manifestations [22]. These capabilities are a result of the complex development of human tumors, and each capability plays a specific role in promoting the growth, advancement, and survival of tumors and their cells [23]. This is crucial for developing targeted and effective treatments.

The analysis revealed that the distinctive characteristics, when considered alone, are inadequate to tackle the intricacies of cancer development. This refers to the specific molecular and cellular mechanisms that allow preneoplastic cells to evolve and acquire abnormal phenotypic capabilities as they develop into tumors and progress into malignancy. As a result, a new concept called “enabling characteristics” was introduced [24]. These characteristics arise because of the abnormal state of neoplasia, providing the mechanisms by which cancer cells and tumors can adopt these functional traits. The acknowledgment of the tumor microenvironment (TME) stems from the understanding that cancer cells enlist a variety of normal cell types to assist in acquiring characteristic functions. TME is a complex and diverse environment that includes various types of cells, such as cancer cells, cancer stem cells, and different stromal cell types. It encompasses both the transformed tissue and the surrounding stroma. The TME is recognized as playing a crucial role in the development and progression of tumors [25,26], making it a promising target for cancer therapy.

**Figure 1:**
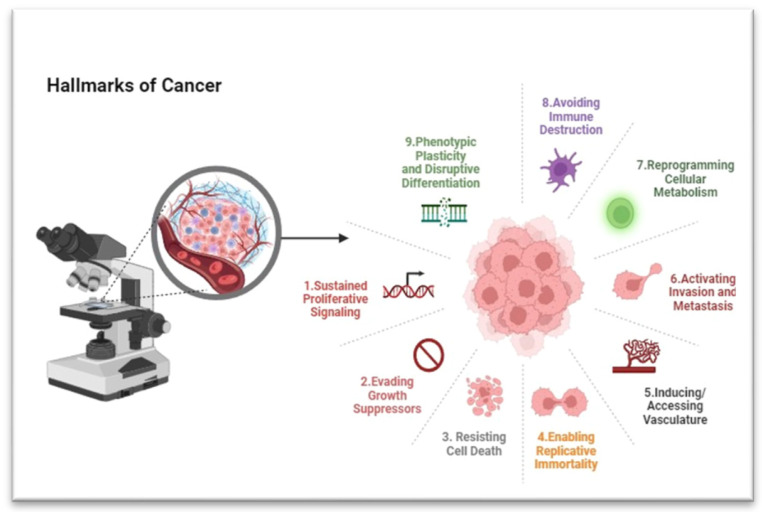
The hallmarks of cancer. Generated by BioRender https://www.biorender.com/, accessed on 29 March 2024).

So far, a total of nine hallmarks have been explained (Figure 1). These include the ability to keep cell division signals going, avoid growth inhibitors, avoid cell death, make an unlimited number of copies, start invasion and spread to other parts of the body, change cellular metabolism, get around the immune system, and stop cells from differentiating normally.

### 3.1. Sustaining Proliferative Signaling

Generally, healthy cells require mitogenic growth signals (GS) in order to shift from a dormant state to an actively proliferative state. The signals encompass diffusible growth factors, constituents of the extracellular matrix, and molecules that promote cell-to-cell adhesion and interaction [27]. Normal tissues carefully regulate the generation and release of these growth signals, maintaining a balance in the number of cells and ensuring the structure and function of the tissue [28]. Tumor cells demonstrate a significant deviation from the typical behavior of normal cells, characterized by a reduced dependence on external signals for growth. This autonomy interrupts a crucial homeostatic mechanism that is responsible for controlling the appropriate functioning of various cell types within a tissue [29]. Within cancer cells, there are significant changes in the growth and signaling pathways that allow them to grow and multiply independently. There are three main molecular strategies that can be used to achieve this autonomy: modifying the growth signals that are outside of the cell, modifying the transcellular transducers that are responsible for transmitting these signals, and modifying the circuits and signaling molecules that are inside the cell [30].

#### 3.1.1. Alteration of Extracellular Growth Signals

Cancer cells can exhibit autocrine stimulation, whereby they are capable of growing without relying on external growth signals (heterotypic signaling). They achieve this by emitting their own growth signals and reacting to them by expressing specific receptors, which generates a positive feedback loop in the signaling process [31]. Instances of this occurrence can be seen in glioblastomas, which secrete Platelet-Derived Growth Factor (PDGF), and sarcomas, which produce Tumor Growth Factor α (TGF α) [32].

#### 3.1.2. Alterations in Transcellular Transducers Responsible for Growth Signals

Cell surface receptors that transmit growth-stimulating signals into the cell interior can undergo modifications in different ways. An example of a mechanism is the excessive production of growth factor receptors, particularly those that have tyrosine kinase functions [33]. An instance of this is the increased expression of the epidermal growth factor receptor (EGF-R/erbB) in tumors located in the stomach, brain, and breast [34]. As shown by the fact that it lacks a large part of its cytoplasmic domain [32,35], the EGF receptor is an example of how structural changes can affect cell surface receptors [29,36].

#### 3.1.3. Alteration of Intracellular Circuits and Cytosolic Signaling Molecules

The development of acquired growth signaling autonomy requires a multitude of intricate steps. These steps entail modifications in the cytoplasmic circuitry located deeper within the cell, specifically in the SOS-Ras-Raf-MAPK cascade. Ras proteins with mutations are present in 25% of human tumors, and these proteins release signals that encourage cell proliferation without being subject to typical upstream controls [31]. Various types of tumors exhibit somatic mutations, indicating that growth factor receptor-mediated signaling pathways remain active [37]. For example, more than 40% of human melanomas exhibit activating mutations in the B-Raf protein, resulting in persistent signaling through the Raf mitogen-activated protein (MAP) kinase pathway [38]. When negative feedback loops do not work right, as they do in the Ras oncoprotein and the phosphatase and tensin homolog deleted on chromosome ten (PTEN) phosphatase, proliferative signaling can become stronger. When PTEN is not expressed in human tumors, the phosphoinositide-3-kinase (PI3K) signaling pathway becomes more active. This makes the tumor grow in a number of cancer models [39].

### 3.2. Evading Growth Suppressors

Normal cells maintain a balance of proliferative signals through the use of tumor suppressor genes (TSGs). These genes act as integral components in the cell’s regulatory mechanisms, similar to braking systems. The retinoblastoma protein pathway (pRb), along with its two counterparts, p107 and p130, plays a vital role in this regulatory framework. It combines signals from various sources outside and inside the cell and, as a result, determines whether a cell should continue with its growth and division process [40]. Also, a p53 protein-based intracellular monitoring system checks the health of the cell before letting it grow and divide. Some substances, such as soluble growth inhibitors and extracellular matrix components, stop cells from multiplying. These signals, along with the signals cells receive through their surface receptors and their own internal signaling pathways [41], keep healthy tissues at rest or in a state of specialization. Cancer cells, on the other hand, elude these signals that inhibit cell growth by interfering with the pRb pathway. This interference renders them unresponsive to these signals and allows them to multiply through E2F transcription factors. The regulation of the pRb pathway is influenced by external factors such as Transforming Growth Factor β (TGF β), a widely recognized antigrowth factor that hinders the progression of the cell cycle [42]. Loss of tumor suppressor function in human tumors can happen in a number of ways, such as when they stop responding to TGF β, obtain bad receptors, or obtain mutations in downstream targets like Smad4 and p15INK4B. Additionally, certain tumors caused by DNA viruses can disable the pRb protein by attaching viral oncoproteins to it [43].

### 3.3. Resisting Cell Death

The proliferation of tumor cell populations is not only determined by the rate of cell division but also by the rate of cell elimination [33]. One significant obstacle to abnormal cell growth is the presence of inherent cellular processes that can trigger cell death [44], specifically apoptosis, autophagy, and necrosis. On the other hand, there is growing evidence indicating that certain mechanisms may unexpectedly promote tumor survival and progression [44,45], as explained below.

#### 3.3.1. Apoptosis

Apoptosis, a fundamental mechanism found in almost all cell types in the body, occurs in response to various physiological signals. This highly coordinated program lasts for a duration of 30–120 min and consists of a series of clearly defined steps. These steps include the disruption of cellular membranes, the breakdown of structures within the cytoplasm and nucleus, the expulsion of cytosol, the degradation of chromosomes, and the fragmentation of the nucleus. Usually, in a span of 24 h, adjacent cells in the tissue consume the shriveled remains, causing them to eventually vanish [46].

The apoptotic machinery consists of two primary categories: sensors and effectors. Sensors monitor the extracellular and intracellular environments, evaluating conditions that determine the survival or death of a cell. They respond to various irregularities like DNA damage, oncogene-induced signaling imbalances, insufficient survival factors, or hypoxia. The Bcl-2 family members, such as Bax and Bak, have either proapoptotic or antiapoptotic roles. They function as sensors for the integrity of mitochondria and control the signaling of mitochondrial death. On the other hand, the p53 tumor suppressor protein triggers apoptosis when there is DNA damage. Together, these sensors have a regulatory influence on the effectors that cause apoptotic death [47].

The ultimate effectors of apoptosis are intracellular proteases known as caspases. Initiating a cascade involving a dozen or more effector caspases, they execute the death program by selectively breaking down subcellular structures, organelles, and the genome [21,48]. Cancer cells try to avoid apoptosis in several ways, but the most common way is to lose a proapoptotic regulator, which usually happens when the p53 tumor suppressor gene is mutated. Without p53, a key part of the DNA damage sensor is missing, which makes it harder for the apoptotic effector cascade to start [49]. Additionally, they may downregulate proapoptotic factors such as Bax and Bak [21]. Tumors can employ alternative strategies to attain similar outcomes, such as upregulating the expression of antiapoptotic regulators like Bcl-2 and Bcl-xL or enhancing survival signals like insulin-like growth factor 1/2 (IGF-1/2.) [49].

#### 3.3.2. Autophagy

Autophagy, a crucial cellular response comparable to apoptosis, becomes prominently activated under specific conditions of cellular stress, particularly when confronted with nutrient deficiency. This programmed process allows cells to disassemble key cellular organelles such as ribosomes and mitochondria, facilitating the recycling of the resulting catabolites. This, in turn, generates essential metabolites and nutrients vital for cell survival and growth, especially when external resources are limited [30,49]. While autophagy primarily serves as a survival mechanism, extreme nutrient deprivation or acute cellular stress can lead to hyperactivation, pushing a cell beyond a reversible point. In such instances, the cell experiences a decline in organelle levels below the critical threshold for viability, resulting in “autophagy-associated” cell death [49].

Researchers have found that mice with inactivated alleles of the Beclin-1 gene are more likely to get cancer. The Beclin-1 gene is an important part of the autophagy machinery. This suggests that autophagy induction may act as a protective barrier against tumorigenesis, operating independently or in conjunction with apoptosis. On the contrary, conditions like nutrient deprivation, radiotherapy, and specific cytotoxic drugs can induce heightened levels of cytoprotective autophagy in cancer cells. Surprisingly, this impairs the effectiveness of stress-inducing treatments rather than enhancing them [49,50]. Furthermore, severely stressed cancer cells have demonstrated the ability to enter a state of reversible dormancy through autophagy, potentially allowing the persistence and subsequent regrowth of some late-stage tumors after treatment with potent anticancer agents [50]. In summary, depending on the physiological state of a neoplastic cell, autophagy has a role in the prevention of early tumor development versus the maintenance and metabolic adaptation of established and metastasizing tumors through autophagy-associated cell death [27].

#### 3.3.3. Necrosis

Necrosis is a planned way for cells to die that is controlled by cellular regulators and effectors. It can be caused by a lack of oxygen and energy, a virus infection, or inflammation [28]. Cells undergoing necrosis undergo swelling and eventual bursting, releasing proinflammatory signals into the surrounding tissue microenvironment. This leads to the recruitment of inflammatory cells from the immune system, which, paradoxically, can actively support tumor development by promoting angiogenesis, cancer cell proliferation, and invasiveness [51]. Consequently, while necrotic cell death may initially appear as a counterbalance to cancer-associated hyperproliferation, its ultimate consequences may be more detrimental than advantageous [31].

### 3.4. Enabling Replicative Immortality

The linear alignment of mammalian chromosomes poses a natural barrier to continuous cell division, primarily because of the presence of telomeres situated at the chromosome ends. The telomeres serve as a mechanism for keeping track of the number of cell generations a lineage has gone through. They accomplish this by gradually reducing their length during each cycle of cell division. The telomeres, located at the ends of each chromosome, are made up of multiple repeating copies of a specific hexanucleotide DNA sequence. These telomeres interact with a specific group of DNA-binding proteins [30,52].

Significantly, when the number of telomere repeats falls below a particular threshold, a signaling mechanism is activated. The activation of the p53 tumor suppressor protein can result in either cell cycle arrest or apoptosis [30]. Tumor cells exhibit traits of immortality, suggesting that the ability to replicate indefinitely is a crucial characteristic for the advancement of malignancy [29]. The ability of cells to maintain telomeric DNA at lengths long enough to fend off senescence or apoptosis and ensure their immortality is what ultimately causes tumor formation. The more common method of accomplishing this is by increasing telomerase expression or, less frequently, by using an alternative recombination-based mechanism to preserve telomeres [49].

### 3.5. Inducing or Accessing Vasculature

Tumors, like regular organs, require the continuous provision of oxygen, glucose, and other nutrients, as well as a means of disposing of metabolic waste, in order to maintain the survival and growth of cells. Tumor-associated vasculature is responsible for fulfilling this role. The growth of cancer cells is impeded when their ability to obtain nutrients from the bloodstream decreases, usually happening when the nearest capillary is more than 200 μ away [30,53].

Angiogenesis, the process of generating new blood vessels, is commonly stimulated and highly beneficial for various types of tumors [30,54]. Angiogenesis is temporarily activated during natural processes such as wound healing and female reproductive cycling. However, in tumor progression, there is almost always a persistent activation of an “angiogenic switch”. The continuous stimulation triggers the activation of previously inactive vessels, facilitating the continuous formation of new vessels. This process supports the continuous neoplastic growth [49].

Cells located at the maximum distance for diffusion from the nearest capillary initiate different stress–response mechanisms, with hypoxia-inducible transcription factors (HIF) being the most notable. HIF controls a variety of genes, including those that directly or indirectly promote the growth of new blood vessels (angiogenesis) and other abilities to adapt to stress [30,55]. The blood vessels that develop within tumors as a result of continuously activated angiogenesis are usually abnormal.

The neovasculature of tumors is distinguished by the early development of capillary sprouting, excessive and convoluted branching of blood vessels, distorted and enlarged vessels, microhemorrhages, increased permeability, and abnormal rates of endothelial cell growth and cell death [49]. Tumors exhibit regions of hypoxia and acidosis due to disorganized blood circulation. These stressful conditions have multiple consequences, such as diminishing the effectiveness of therapy and promoting the expansion of resistant clones [45]. Although chronic angiogenesis is a characteristic feature of most solid tumors, certain tumors may employ an alternative approach to reach the blood vessels. Some cancers can take advantage of their characteristic abilities to invade and spread to hijack the blood vessels of normal tissues [30].

### 3.6. Activating Invasion and Metastasis

Cancer cells exhibiting a high-grade status acquire invasive and migratory characteristics. These cells have the ability to invade nearby tissues and enter blood and lymphatic vessels (intravasation) through aggressive growth programs. These vessels then act as conduits for the spread of cancer cells to nearby and distant anatomical locations. The lymphatic system transports cancer cells from the tissue to the lymph nodes, where they can form lymph node metastases. These metastatic colonies can serve as staging areas, enabling further dissemination by entering the bloodstream [30,49,56].

The development of metastases is responsible for 90% of human cancer fatalities [29]. For accurate control of invasion and metastasis, natural cellular processes and help from nearby accessory cells in the tissue microenvironment must work together in a complex way. One important mechanism that regulates cancer cells involves the activation of a developmental program called the epithelial–mesenchymal transition (EMT) in epithelial cancer cells, specifically carcinomas. This process is strongly linked to the movement of cells and the invasion of tissues, which is similar to the events seen during normal organ development [30,56].

### 3.7. Reprogramming Cellular Metabolism

The metabolic patterns observed in cancer cells closely resemble those of actively dividing normal cells rather than exhibiting a distinct characteristic unique to neoplasia [30]. Observations suggest that cancer cells have an increased ability to take in glucose and undergo glycolysis, even when there is enough oxygen for oxidative phosphorylation to occur. Aerobic glycolysis produces both ATP and numerous vital cellular components necessary for growth and division. The shift from oxidative phosphorylation to aerobic glycolysis in cancer cells is not a binary switch; rather, cancer cells persistently utilize both oxidative phosphorylation and glycolysis, albeit at different rates. The relative locations of these metabolic pathways can change over time and show differences between cancer cells in certain areas of a tumor and in different tissue microenvironments [30,57]. Using positron emission tomography (PET) scanners has helped explain why cancer cells use more glucose even when oxygen is present. Increased levels of glucose transporter (GLUT) proteins have been detected in various cancers, and their presence has been associated with unfavorable outcomes in specific types of cancer [45].

Glutamine is an essential energy source and a precursor to lipids and amino acids found in the bloodstream. Glutamine is typically anticipated to augment and bolster glucose in providing energy and biomaterials for the growth and proliferation of cancer cells. Nevertheless, when there is a lack of glucose, the absorption and processing of glutamine might be able to make up for it [30].

Lactate serves as a crucial participant in metabolic fueling, occupying the third position. In cancer cells experiencing a lack of glucose, extracellular lactate is brought in using specific transporters. This lactate serves as an essential source of fuel for ATP production and the synthesis of biomaterials. Cancer-associated fibroblasts (CAFs) also utilize lactate for energy, benefiting from this metabolic synergy [30,58].

### 3.8. Avoiding Immune Destruction

Cancerous tumors must develop strategies to avoid detection by the immune system, thus preventing the elimination of abnormal cells that are multiplying in a potentially cancerous manner [30]. When a person with a strong immune system encounters a highly reactive tumor, their innate immune system is activated in the best possible way, leading to the quick elimination of the tumor through immune-boosting responses. However, in individuals with weakened immune systems or when facing tumors with a lower immune response, complete eradication may not be achieved. This enables certain cancer cells to survive while being monitored by the immune system, resulting in a prolonged period of balance. During this stage, the tumor undergoes alternating periods of gradual expansion, immune-mediated eradication, and subsequent regrowth. The equilibrium state can endure for an individual’s entire lifespan, resembling elimination, or it can be disturbed by alterations in the tumor or immune system, ultimately resulting in the tumor evading immune control [59]. Conversely, the immune response to around 20% of human tumors caused by viruses exhibits unique characteristics. Oncogenic viruses produce foreign antigens, such as oncoproteins, that are responsible for causing cell transformation. The immune system is unable to recognize these antigens, which results in a lack of tolerance. This stimulates both humoral and cellular immune responses that can eliminate virus-infected precancerous cells and eradicate early-stage tumors. The ability of virus-transformed cells to resist the immune system’s destruction and progress into cancer, however, emphasizes the immune-evasive traits that tumor viruses have developed or that virus-transformed cancer cells have chosen to possess [30,60].

### 3.9. Phenotypic Plasticity and Disrupted Differentiation

During organ development, cells transform into specialized tissues through a process known as differentiation, adopting specific roles crucial for maintaining balance in the body. This includes terminal differentiation, where progenitor cells cease their growth. This natural mechanism typically hinders excessive cell proliferation and serves as a barrier against the abnormal growth observed in cancer. Recent findings indicate that a key role in cancer development involves unlocking the limitations on the ability of cells to adapt their characteristics. This unlocking enables cancer cells to evade or break free from the usual process of terminal differentiation [61]. This plasticity can manifest in various ways. For example, cancer cells emerging from a normal cell that had attained a fully differentiated state may backtrack by dedifferentiating to progenitor-like cell states. On the other hand, neoplastic cells originating from a progenitor cell programmed for end-stage differentiation may bypass this process, maintaining cancer cells in a partially differentiated, progenitor-like state. Another possibility is trans-differentiation, where cells initially committed to one differentiation pathway switch to an entirely different developmental program. This results in the acquisition of tissue-specific traits not initially determined by their normal cells of origin [62,63].

## 4. Bioactive Compounds in Food with Anticancer Activity

### 4.1. Thymoquinone

Thymoquinone (TQ) is a non-toxic monoterpenoid compound, chemically identified as 2-Isopropyl-5-methyl-1,4-benzoquinone, and is derived from the essential oil of *Nigella sativa*, commonly known as black seed [64,65]. TQ has a broad spectrum of pharmacological activities, such as anti-inflammatory, immunomodulatory, hepatoprotective, antihistaminic, antimicrobial, antidiabetic, and antihypertensive effects, as well as significant anticancer efficacy [66,67,68]. TQ has demonstrated anticancer efficacy against various types of cancer, including breast cancer [68], prostate cancer [69], gastric cancer [70], and bladder cancer [71].

TQ exerts its anticancer effects through various mechanisms, targeting multiple hallmarks of cancer. Some of these mechanisms include the modulation of epigenetic machinery, alteration of non-coding RNA gene expression, and influence on various biological pathways related to apoptosis, cell proliferation, cell cycle regulation, hypoxia, and cancer metastasis [72,73]. The induction of apoptosis by TQ is attributed to multiple mechanisms, including the activation of caspases, downregulation of oncogenes, and inhibition of nuclear factor kappa-light-chain-enhancer of activated B cells (NF-κB) [74,75]. In an in vivo study, TQ demonstrated cell inhibition and increased caspase-3 activity to induce apoptosis. Specifically, oral administration of TQ (20 mg/kg) enhanced the effectiveness of cisplatin in hepatocellular carcinoma (HCC) treatment by regulating the GRP78/CHOP/caspase-3 pathway [76]. Additionally, in breast cancer treatment, a combination of TQ and paclitaxel significantly increased apoptotic and necrotic cell death in T47D cells and induced autophagy in MCF-7 cells [77].

TQ has also been shown to alter cell proliferation and induce cell cycle arrest. Treatment of papilloma (SP-1) cells with TQ-induced G0/G1 cell cycle arrest was associated with a significant increase in the expression of the cyclin-dependent kinase inhibitor p16 and a decrease in cyclin D1 protein levels. In spindle (I7) cells, TQ-induced growth inhibition occurred through G2/M cell cycle arrest, which was linked to an increased expression of the tumor suppressor protein p53 and a reduction in cyclin B1 protein expression [78]. Furthermore, TQ induces G1 phase cell cycle arrest in human breast cancer, colon cancer, and osteosarcoma cells by inhibiting the activation of cyclin E or cyclin D and upregulating the cyclin-dependent kinase (CDK) inhibitors p27 and p21 [79]. In esophageal cancer, TQ induces G2/M phase cell cycle arrest by increasing the levels of p21 and p53 while significantly decreasing the expression of cyclin A, cyclin B1, and cyclin E [80].

### 4.2. Resveratrol

Resveratrol, known as 3,5,4′-trans-trihydroxystilbene, stands as a polyphenolic phytoalexin within the stilbene category. Originating primarily from grape skin and seeds, this natural dietary component is present not only in wines but also in a diverse range of plant-based foods, notably including peanuts, berries, and tea [81]. Resveratrol was initially identified and extracted from white hellebore by the Japanese researcher Takaoka in 1939. Over 70 plant species engage in the synthesis of resveratrol as a response to various stimuli such as infection, stress, injury, bacterial or fungal infections, and exposure to UV irradiation [82]. The production of this molecule in plants involves the catalytic action of resveratrol synthase in the phenylpropanoid pathway, demonstrating a pathway analogous to that of flavonoids [83]. Resveratrol is characterized by the presence of two phenol rings (monophenol and diphenol) connected through a double styrene bond, existing in both cis and trans isomeric forms. Notably, trans-resveratrol is observed to be the more prevalent and stable natural configuration [84]. With three hydroxyl groups, this molecule actively participates in free radical scavenging and metal chelation. Its hydroxyl groups, benzene ring, and C–C double bond enable multiple interactions with macromolecules [85,86].

In recent years, resveratrol has attracted widespread attention motivated by the finding that dietary patterns rich in resveratrol substantially diminish all-cause mortality [87,88,89]. Numerous epidemiological studies have investigated the correlation between resveratrol consumption and human health. The observed benefits linked to resveratrol consumption have encouraged extensive research exploring additional health outcomes and the underlying molecular mechanisms [88]. Resveratrol displays a broad spectrum of biological properties, including anti-glycation, antioxidant, anti-inflammatory, neuroprotective, anti-cancer, and anti-aging activities [87]. Furthermore, studies have indicated its immunomodulatory effects, cardiovascular protective mechanisms, protective efficacy against diverse liver diseases, and anti-diabetic potentials [88].

Rats exhibited high tolerance to resveratrol, with no observable toxicological effects noted at doses ranging from 700 to 1000 mg/kg body weight/day [90]. Short-term human studies utilizing doses of up to 2 g/day revealed no significant side effects [91]. Notably, in clinical trials, resveratrol demonstrated safety and favorable tolerance, with no notable reports of toxicity, even at doses as high as 5 g/day [92].

Resveratrol stands out as a promising complement in the therapeutic and preventive strategies against various cancers, including cervical, hepatic, prostatic, cerebral, thyroidal, ovarian, esophageal, gastric, bronchial, pulmonary, cutaneous, and head and neck malignancies [93]. Its natural origin enhances its effectiveness, while its safety and cost-effectiveness make it an attractive alternative to conventional cancer treatments [93]. Research has examined various mechanisms to explain the impact of resveratrol on diverse cancer cell types, namely cell apoptosis, antiproliferation, anti-inflammation, signaling pathways related to growth factors and receptor tyrosine kinases, and signal transduction by the growth factor β [94,95].

The anticancer mechanisms of resveratrol involve targeting key hallmarks of cancer, including the inhibition of three critical glycolytic enzymes: hexokinase (HK) and phosphofructokinase (PFK1) [96]. In many neoplastic cells, HK II is overexpressed and plays a crucial role in supporting the proliferation and survival of tumor cells [97]. Resveratrol has been shown to indirectly suppress HK II by reducing the translocation of HK II to the outer mitochondrial membrane (OMM) via the inhibition of the receptor tyrosine kinase (RTK)/phosphoinositide-3-kinase (PI3K)/AKT/mammalian target of rapamycin (mTOR) pathway in a range of cancer cell types [98]. Detaching HK II from the mitochondria could make the mitochondria more vulnerable to damage caused by cisplatin, leading to a marked increase in apoptotic cell death [99]. The inhibition of PFK1, another key glycolytic enzyme, has been found to be a key factor in inducing cell death in human breast cancer cell lines [100]. Resveratrol was found to reduce viability, glucose consumption, and ATP content in the human breast cancer cell line MCF-7 by inhibiting PFK1 [101].

The anti-proliferative effects of resveratrol in tumor cells were shown to be mediated through cell cycle arrest by altering the activity of protein kinase complexes, which consist of cyclin-dependent kinases (CDKs) and their associated cyclins [96]. In colon cancer cells, resveratrol showed down-regulation of the cyclin D1/CDK4 complex [102]. Resveratrol can induce G1 phase arrest by decreasing the protein levels of cyclin D2, cyclin E, and CDK2, CDK6 through the inhibition of transcription factors like nuclear factor-kappa B (NF-κB) and activator protein-1 (AP-1) [103]. Resveratrol can also induce G2 phase arrest by inhibiting CDK7 and CDK2 kinases in colon carcinoma cells [104].

### 4.3. Piperine

Piperine is an alkaloid with the IUPAC name [1-[5-[1,3-benzodioxol-5-yl]-1-oxo-2,4-pentadienyl] piperidine]. It is the principal bioactive compound found in *Piper nigrum* and *Piper longum* [105]. Besides being found in Piper species, piperine is also present in the leaves of *Rhododendron faurie* [106], *Vicoa indica*, seeds of *Anethum sowa*, *Fructus piperis longi* [107], and the bark of *Careya arborea* [108]. Piperine was first extracted from the dried fruit of pepper in 1820. It forms a yellow crystalline solid with a molecular weight of 285.33 g/mol and a melting point of 128–130 °C [109]. Piperine is water-insoluble and has a weakly basic nature. It is initially tasteless but leaves a burning sensation shortly after [110].

Piperine provides a broad spectrum of health benefits, including antioxidant, antihypertensive, antidiarrheal, anti-inflammatory, hepatoprotective, antidepressant, antibacterial, and immunomodulatory effects, as well as significant anticancer properties. Many of these benefits have been validated through both in vivo and in vitro studies [110,111].

In colon cancer cell lines, piperine at concentrations of 75–150 μM inhibited the growth of HT-29 colon carcinoma cells by inducing G1 phase cell cycle arrest. This was linked to decreased levels of cyclin D1 and D3, along with their associated CDK4 and CDK6. Piperine also triggered the production of hydroxyl radicals and apoptosis [112]. Piperine has been shown to inhibit osteosarcoma cell proliferation in a dose- and time-dependent manner. This inhibition involves G2/M phase cell cycle arrest and a reduction in the migration and invasion of HOS and U2OS cells. The mechanisms include increased expression of tissue inhibitors of metalloproteinases (TIMP-1 and TIMP-2) and decreased levels of matrix metalloproteinases (MMP-2 and MMP-9) [113]. Piperine was also found to inhibit the growth of triple-negative breast cancer (TNBC) cells. It achieved this effect by inducing G2 phase cell cycle arrest, reducing the expression of proteins associated with the G1 and G2 phases, increasing the expression of p21 (Waf1/Cip1), and suppressing survival-promoting Akt activation. These actions collectively lead to caspase-dependent apoptosis [112]. Jafri and colleagues demonstrated that piperine induces apoptosis in human cervical adenocarcinoma cells in a dose-dependent manner. This is achieved through increased generation of reactive oxygen species (ROS), nuclear condensation, disruption of mitochondrial membrane potential, DNA fragmentation, and, ultimately, activation of caspase-3 [114]. In research conducted by Si et al., treatment with 8 µM piperine resulted in a marked decrease in the viability of ovarian cancer cells after 48 h while having minimal impact on normal ovarian cells [115].

In a study conducted by Sriwiriyajan et al., piperine was shown to inhibit proliferation in rats with N-nitroso-N-methylurea-induced mammary tumorigenesis. This effect was associated with downregulation of E-cadherin (E-cad), estrogen receptor (ER), matrix metalloproteinases 2 and 9 (MMP-2 and MMP-9), vascular endothelial growth factor (VEGF), and c-Myc, while simultaneously upregulating p53 [116]. In a murine lung metastasis model evaluating piperine’s anti-metastatic effects, it significantly reduced both the size of tumor nodules and the levels of uronic acid and hexosamine, which are involved in the metastasis pathway [117].

### 4.4. Genistein

Genistein (C15H10O5), also known as [4,5,7-trihydroxyisoflavone], is the most prominent isoflavone present in soy products [118]. It can also be found in plant foods, including alfalfa and clover sprouts, barley meal, broccoli, cauliflower, sunflower seeds, caraway, and clover seeds [119]. Genistein is a biologically active isoflavone with several significant health benefits. These include reducing body mass and fat tissue, which lowers the risk of cardiovascular diseases [120]. Genistein is classified as a phytoestrogen with a diphenol structure similar to endogenous estrogen. This resemblance enables genistein to bind to estrogen receptor subtypes α and β, which may explain its potential in preventing osteoporosis, alleviating post-menopausal issues, and playing a role in the prevention of hormone-related cancers [119,120,121].

Numerous studies have explored genistein’s anticancer effects. Its mechanisms of action include influencing inflammation, cellular proliferation, and epigenetic modifications [121]. Furthermore, genistein has been demonstrated to inhibit both angiogenesis and metastasis [120].

A study assessing the effects of genistein on TNBC cells using the MDA-MB-231 cell line treated for 72 h with different concentrations of genistein (5, 10, and 20 μM) found that genistein downregulated the expression of cyclin B1, Bcl-2, and Bcl-xL. This downregulation, potentially mediated by Nuclear factor kappa B (NF-κB) through the Notch-1 signaling pathway, is linked to the inhibition of cell proliferation and the induction of apoptosis in tumor cells [122]. Another study on prostate cancer cells revealed that genistein directly inhibits the Akt and NF-κB pathways, leading to the activation of apoptosis [123]. Treatment with genistein in HCT-116 and SW-480 human colon cancer cells (25–100 μM for 48 h) exhibited growth inhibition and dose-dependent promotion of apoptosis. Genistein induced G2/M phase cell cycle arrest in a p53-dependent manner and activated the ATM/p53 pathway, along with p21 and GADD45a. Additionally, it downregulated CDK1 and cdc25A, which are key regulators of the cell cycle and apoptosis [124]. Gu and colleagues used the MHCC97-H hepatocellular carcinoma (HCC) cell line to validate the anti-metastatic activity of genistein. Treatment with genistein (at 10 and 20 μM) induced cell cycle arrest at the G2/M phase. Additionally, genistein was found to block focal adhesion kinase (FAK), a cytoplasmic tyrosine kinase associated with cell growth, adhesion, and motility. By inhibiting FAK overexpression, genistein reduces the metastatic potential of HCC. Furthermore, it hinders HCC progression by inducing cell cycle arrest and apoptosis [125].

### 4.5. Allicin

Allicin, or diallyl thiosulfinate, is a naturally occurring volatile compound characterized by its sulfur content. It is a defensive compound responsible for the characteristic smell and taste of freshly cut or crushed garlic [126]. It is found in white garlic (*Allium sativum* L.) as well as in other *Allium* species, including elephant garlic (*A. ampeloprasum* L.), wild garlic (*A. ursinum* L.), field garlic (*A. vineale* L.), and alpine leek (*A. victorialis* L.) [127]. Allicin is a colorless oil that has poor solubility in water, which accounts for its ability to pass easily through cell membranes [128]. Allicin is a molecule that is both unstable and short-lived. After its formation, it rapidly breaks down into various secondary compounds, influenced by factors such as temperature and pH [129]. Besides its contribution as a promising anticancer agent, allicin has demonstrated several therapeutic properties, including cardiovascular protection, blood pressure-lowering, antioxidant, antimicrobial, anti-asthmatic, and immunoregulatory benefits [127].

Research has investigated the anticancer properties of allicin, highlighting its ability to target various cancer hallmarks. For example, allicin has been shown to deplete redox defenses, leading to apoptosis. In a study by Miron and colleagues, the effect of allicin at 5 μM was assessed on human promyelocytic leukemia-derived HL60 cells and human myelomonocytic U937 cells. Allicin treatment resulted in the oxidation of -SH groups in various biomolecules (such as cysteine, glutathione, proteins, and peptides), altering the intracellular redox balance. This glutathione depletion caused cell death and exhibited overall anti-proliferative activity [130]. Allicin has also been shown to induce apoptosis via NF-E2-related factor-2 (Nrf2). In studies with colon cancer cell lines (such as HCT-116, LS174T, HT-29, and Caco-2), allicin treatment resulted in increased hypodiploid DNA content, involvement of Bcl-2/Bax, and release of cytochrome c into the cytosol [131]. Autophagy has been recognized as a mechanism of cell death in human thyroid cancer cells SW1736 and HTh-7, particularly when treated with a combination of 10 μM allicin and either 10 μM cisplatin or carboplatin [132]. In a study by Chen and colleagues, allicin (40 µM) was found to inhibit the proliferation and invasion of cholangiocarcinoma (CCA) cells by targeting STAT3, a key transcription factor involved in cell proliferation [133]. A different study demonstrated that allicin, at concentrations of 15.0 and 20.0 µM, exhibits anticancer effects in lung adenocarcinoma cells (A549 and H1299 cells) by targeting the PI3K/AKT pathway. These effects involve the suppression of cell proliferation, invasion, and metastasis [134]. Allicin can also inhibit angiogenesis in lung cancer cells (A549) by decreasing VEGF-A protein expression, suppressing VEGF-A gene expression, targeting the HIF pathway, and enhancing immune system activity [135].

### 4.6. Epigallocatechin Gallate

Epigallocatechin gallate (EGCG) is the most prevalent bioactive polyphenol found in green tea. It is also present in black and oolong teas and can be found in small amounts in various fruits and vegetables. It is a flavonoid with numerous health benefits, extensively studied for its effects on cardiovascular disorders, diabetes, and cancer, which are attributed to its polyphenolic catechin content [136]. Among catechins, EGCG demonstrates the strongest anti-proliferative and pro-apoptotic effects against cancer cells [136]. A study on colon adenocarcinoma cells (COLO205) found that EGCG, at concentrations ranging from 5 to 40 µg/mL, impacted chromosome instability, triggered apoptosis, and inhibited cell division. Interestingly, the same study revealed that EGCG also offered protective effects to normal cells at these concentrations [137]. In a study by Jiang and colleagues, the treatment of nasopharyngeal carcinoma with 40 µM EGCG induced apoptosis through the downregulation of Sirtuin 1 (SIRT1) [138]. In MCF-7 breast cancer cells, treatment with EGCG at 30 µmol/L significantly inhibited cell proliferation and triggered apoptosis, potentially through modulation of the P53/Bcl-2 signaling pathway [139]. On the other hand, in lung adenocarcinoma, a low dose of EGCG (0.5 µM) amplified the toxicity of Doxorubicin (10 µM) and demonstrated antineoplastic effects mediated by oxidative damage, likely by modulating redox signaling in A549 cells [140]. In terms of inhibition of angiogenesis, a study involving head, neck, and breast carcinomas found that EGCG at 30 µg/mL reduced VEGF production by interfering with epidermal growth factor receptor (EGFR)-related pathways [141].

### 4.7. Curcumin

Curcumin, also known as diferuloylmethane, is the primary natural polyphenol found in the rhizome of *Curcuma longa* (turmeric) and other *Curcuma* species. Isolated for the first time in 1815, it is renowned for its role in addressing inflammation, arthritis, metabolic syndrome, liver disease, obesity, neurodegenerative diseases, and, most notably, various cancers [142]. Curcumin, with a molecular weight of 368.38, is insoluble in water but can dissolve in highly acidic or alkaline solvents, as well as in ethanol and DMSO. It is a bright orange-yellow crystalline compound, which makes it useful as a food colorant [143]. Curcumin demonstrates anti-cancer properties by affecting various biological pathways, including mutagenesis, oncogene expression, cell cycle regulation, apoptosis, metastasis, and angiogenesis. Numerous studies have been conducted to investigate these effects.

In a study on non-small-cell lung cancer (NSCLC), curcumin demonstrated the ability to inhibit cell proliferation, enhance apoptosis, and induce G2/M phase cell cycle arrest. It also promotes ROS production, thereby triggering the DNA damage signaling pathway [144]. In an alternative NSCLC cell model, prolonged administration of low-dose curcumin at concentrations between 0.25 and 0.5 µM led to a substantial reduction in both the invasiveness and metastatic potential of the cancer cells [145]. Shan and colleagues demonstrated that curcumin impedes the phosphorylation of Akt and mTOR, as well as their associated signaling proteins. This inhibition triggers cell cycle arrest across various breast cancer cell lines, including T47D and MCF7 [146]. In the K562 leukemic cell line, curcumin demonstrated a dose- and time-dependent inhibition of cell proliferation and clonogenicity, specifically targeting the Wilms tumor 1 (WT1) protein, which is highly expressed at both the mRNA and protein levels. Additionally, curcumin-induced cell cycle arrest at the G2/M phase [147]. Curcumin has also shown significant anticancer effects against digestive system cancers. For example, in gastric SGC-7901 cells, curcumin-induced apoptosis disrupts mitochondrial membrane potential (MMP) and causes the release of cytochrome c into the cytosol. This effect was accompanied by the downregulation of Bcl-2, upregulation of Bax, and activation of caspase-3 cleavage [148].

### 4.8. Emodin

Emodin, chemically identified as 1,3,8-trihydroxy-6-methylanthraquinone, is a natural anthraquinone derivative found as an active ingredient in the root and rhizome of *Rheum palmatum* [149]. Emodin, a tyrosine kinase inhibitor, has demonstrated multiple biological activities, including anti-inflammatory, antibacterial, and anticancer effects across various cancer types. These include NSCLC, colon cancer, breast cancer, prostate cancer, pancreatic cancer [150]. In NSCLC, emodin primarily acts by modulating the cell cycle, decreasing the proportion of cells in the G2/M and S phases while increasing the number of cells in the G1/G0 phase [151]. In addition, treatment with emodin (5 µM) enhanced the sensitivity of H460 and A549 non-small lung cancer cells to cisplatin by downregulating P-glycoprotein [152]. Han et al. discovered that emodin effectively suppressed the phosphatase activity of phosphatase of regenerating liver-3 (PRL-3) in the DLD-1 colon cancer cell line and dose-dependently inhibited PRL-3-induced tumor cell migration and invasion [153]. In a study involving human breast cancer BCap-37 cells, emodin was found to induce apoptosis by lowering the Bcl-2/Bax ratio and increasing the concentration of cytochrome c in the cytoplasm [154]. Yu et al. reported that emodin triggered apoptosis in prostate cancer LNCaP cells by upregulating caspase-3 and -9 and by increasing the Bax/Bcl-2 ratio [155]. While in pancreatic cancer, emodin exerts its antitumor effects by lowering the levels of X-linked inhibitor of apoptosis protein (XIAP) and suppressing NF-kB signaling [156].

### 4.9. Parthenolide

Parthenolide (PTL) is a naturally occurring sesquiterpene lactone extracted from the feverfew plant (*Tanacetum parthenium*). Numerous studies have highlighted the promising anti-inflammatory and anti-tumor properties of this natural product [157]. Research has focused on how PTL influences a range of cancer-associated signaling pathways and targets involved in cell proliferation. PTL triggers apoptosis through various mechanisms, such as blocking the NF-κB pathway, initiating DNA hypomethylation and histone acetylation, increasing oxidative stress through endoplasmic reticulum or thiol depletion, and activating p53 [158,159]. It has also been demonstrated that PTL significantly inhibits cell growth and induces G0/G1 cell cycle arrest through multiple mechanisms, including transcriptional regulation, modulation of signal transduction pathways, and induction of oxidative stress [159,160]. In a study focused on renal cell carcinoma, PTL was found to inhibit oncogenic traits in 786-O and ACHN cells, reduce cancer cell viability, and suppress mammosphere formation [161]. In another study involving lung cancer cell lines A549 and H1299, PTL significantly impeded cell proliferation and migration by blocking the phosphorylation of insulin-like growth factor 1 receptor (IGF-1R), Akt, and forkhead box O3α (FoxO3α) [162]. Additionally, in cervical cancer, PTL demonstrated potent anticancer activity by reducing HeLa cell viability in a dose-dependent manner. It also induced the production of reactive oxygen species (ROS), leading to a loss of mitochondrial membrane potential [163].

### 4.10. Luteolin

Luteolin, a flavonoid chemically identified as 3′,4′,5,7-tetrahydroxyflavone, is present in a variety of vegetables and fruits such as celery, parsley, broccoli, onion leaves, carrots, peppers, cabbages, and apple skins. It is known for its heat stability, retaining its properties even with cooking [164]. Luteolin has the ability to impact various characteristics of cancer cells. For instance, in HT-29 human colon cancer cells treated with luteolin, IGF-1R signaling was reduced, resulting in the downregulation of both the PI3K/Akt and ERK1/2 pathways [165]. It was also observed that luteolin induces apoptosis in tamoxifen-resistant breast cancer by activating apoptosis-related proteins, including cleaved poly (ADP-ribose) polymerase and cleaved Caspase-7, Caspase-8, and Caspase-9 [166]. Luteolin also enhances the expression of miR-124-3p and activates the death receptor and mitogen-activated protein kinase (MAPK) signaling pathways in glioma [167]. In metastatic human colon cancer, luteolin was shown to decrease the viability and proliferation of SW620 cells. It reduced the levels of the antiapoptotic protein Bcl-2 while increasing the expression of antioxidant enzymes and promoting the proapoptotic proteins Bax and caspase-3 [168].

### 4.11. Quercetin

Quercetin (C_15_H_10_O_7_), chemically known as 3,3′,4′,5,7-pentahydroxyflavone, is a natural flavonoid. It occurs as a glycoside in numerous plants, fruits, and vegetables, including onions, buckwheat, and broccoli. Being lipophilic, quercetin can easily cross cellular membranes and activate various intracellular pathways that contribute to its anticancer effects [169]. Quercetin has been reported to induce apoptosis through both direct activation of the caspase cascade and the mitochondrial pathway. This effect has been observed in various human cell lines, including breast cancer MCF-7 cells [170], leukemia HL-60 cells [171], thymus-derived HPBALL cells [172], and oral squamous carcinoma SCC-9 cells [173]. It has been reported that quercetin increases the expression of the retinoblastoma (Rb) gene, leading to cell cycle arrest in the G2/M or G0/G1 phase in nasopharyngeal carcinoma HK1 and CNE2 cells [174]. Moreover, quercetin induced cell cycle arrest in MCF-7 cells at the S phase in a dose- and time-dependent manner. This arrest was linked to decreased levels of CDK2 and cyclins A and B, alongside increased expression of p53 and p57 [170]. Treatment with quercetin resulted in decreased levels and activity of MMP-2 and MMP-9 in several cancer cell lines, which contributed to its anti-angiogenic effects [169]. In melanoma, quercetin inhibited STAT3 signaling, leading to reduced expression of its downstream target genes, including Mcl-1, MMP-2, MMP-9, and VEGF, which are crucial for cell proliferation, migration, and invasion [175].

### 4.12. Anthocyanins

Anthocyanins are a class of natural flavonoids commonly found in a variety of fruits and vegetables, including berries, pomegranates, and rice, where they exist primarily as glycosides [176]. These compounds are water-soluble pigments that can exhibit different colors—such as red, purple, blue, or black—depending on their pH level [177]. Anthocyanins are involved in various health conditions, such as cardiovascular, neurological, and metabolic diseases. Additionally, they have a crucial role in cancer management, attributed to their antioxidant, anti-inflammatory, anti-invasive, and anti-metastatic properties [178]. Lu et al. reported that anthocyanin-rich blueberry extracts, at concentrations between 6.25 and 100 µg/mL, inhibited lung cancer by reducing the growth of A549 cells [179]. In a separate study on breast cancer, Lage and colleagues found that anthocyanin extracts from dark sweet cherries significantly suppressed cell proliferation and induced apoptosis by inhibiting the Akt/mTOR and Sirt1/survivin pathways. Additionally, these extracts effectively inhibited metastasis by down-regulating the expression levels of Sp1, Sp4, and VCAM-1 [180]. Lopez de Las Hazas et al. observed that anthocyanin extracts from grapes and berries displayed anti-colon cancer effects by inducing apoptosis in HT-29 cells [181]. On the other hand, Mazewski et al. showed that anthocyanin extracts from red grapes and black lentils inhibited angiogenesis by decreasing the expression levels of PVGF [182]. Finally, regarding liver cancer, Bishayee et al. demonstrated that anthocyanins exert anticancer effects in rats by inhibiting proliferation and inducing apoptosis through the regulation of Bax and Bcl-2 expressions [183]. Additionally, Thi and Hwang found that anthocyanins derived from black chokeberry, at concentrations of 100–200 µg/mL, exhibited anticancer activity in SK-Hep1 cells by inhibiting proliferation, adhesion, migration, and metastasis [184]. Table 1 sumarrizes all bioactive compounds from food with their anticancer mechanisms.

## 5. Dietary Management with Cancer Prevention Potential

Prior epidemiological research has indicated that a mere 5–10% of cancers can be directly linked to genetic abnormalities, while the remaining majority are influenced by factors such as diet, environment, and lifestyle. Although modifiable factors can help control most cancers, the global incidence rates of cancer are on the rise [185]. Additionally, the field of human cancer research has shown a significant interest in dietary management. Numerous observational studies have indicated that adopting a healthy and balanced diet can lower the risk of cancer and enhance the effectiveness of cancer treatment [186,187,188].

Several natural food components demonstrated targeted inhibition of cancer cells [189]. A study uncovered that numerous cancer patients opt for complementary and alternative therapies as a means of managing their illness, alleviating its symptoms, and mitigating the adverse effects of cytotoxic drug treatment. Therefore, in order to enhance clinical outcomes, it may be beneficial to incorporate natural or herbal products alongside traditional chemotherapy and cancer treatments. This can help rebalance the body and inhibit the initiation, progression, and metastasis of tumors [190].

### 5.1. Inflammation-Reducing Diet

Chronic inflammation can be attributed to various factors, including the consumption of inflammatory foods. Even at a low level, this can cause significant cellular stress and worsen metabolic disorders, as well as contribute to the development of non-communicable diseases like cancer [191]. A systematic review and meta-analysis examining the relationship between dietary inflammatory index and cancer incidence has found that pro-inflammatory diets are generally linked to a higher risk of cancer compared to anti-inflammatory diets. This supports the notion that an inflammatory diet is a risk factor for cancer [192]. It is generally advised to incorporate anti-inflammatory foods into one’s daily diet, such as tomatoes, green leafy vegetables, fruits like oranges, omega-3 dietary sources like seafood, and healthy nuts like almonds. However, certain foods, like refined carbohydrates, red meat, soda, and fried potatoes, are associated with inflammation and consequently elevate the risk of cancer [193].

Phytochemicals such as plant polyphenols are generally considered to have anticancer, anti-inflammatory, and immunomodulatory effects, which explain their role in human health. The previous studies have contributed to a growing evidence base in the literature that demonstrates the ability of polyphenols to modulate multiple targets of carcinogenesis, linking models of cancer characteristics (i.e., hallmarks and nutraceutical-based targeting of cancer) through direct or indirect interaction or modulation of cellular and molecular targets. This evidence is particularly relevant for lignans, a ubiquitous and important type of dietary polyphenol present in high levels in food sources such as flaxseed [194,195].

### 5.2. Alkaline Diet

A systematic review and meta-analysis examined the correlation between acidosis and the risk of cancer. The findings suggested that a high acid load could be linked to an elevated risk of cancer. The Warburg hypothesis, which states that cellular hypoxia is one of the main causes of cancer, can explain this association. This leads to the substantial production of lactic acid through anaerobic cellular respiration, ultimately resulting in inflammation [187]. Incorporating specific raw foods with a negative dietary acid load, such as garlic, onion, spinach, dates, and oranges, into a healthy diet can enhance blood oxygen saturation at the cellular level. This, in turn, lowers the likelihood of developing cancer [186,191]. Also, earlier studies have shown support for the alkalizing diet hypothesis, which says that a healthy plant-based diet with alkalizing properties may lower the risk of getting breast cancer, prostate cancer, colorectal cancer, and lung cancer [186,196,197].

### 5.3. Ketogenic Diet

A ketogenic diet and other sugar-free high-fat diets are not advisable for cancer prevention or treatment due to their significant amounts of red meat, saturated fat, and processed meat, which elevate the risk of cancer. Additionally, these diets are lacking in vegetables, fruits, legumes, fiber, and whole grains, which are known to provide protection against cancer. In addition, eliminating advantageous sugars from the diet in order to deprive cancer cells of nutrients could also harm and debilitate normal cells, and in certain instances, it may mitigate the advancement of cancer without providing any tangible advantages in terms of treatment results and survival rate [186,198].

## 6. Conclusions

The aim of this review was to examine various bioactive food ingredients for cancer management by targeting the hallmarks of cancer, including sustaining proliferative signaling, evading growth suppressors, resisting cell death, enabling replicative immortality, inducing angiogenesis, activating invasion and metastasis. This review demonstrated the complexity of cancer and the importance of understanding these hallmarks to enhance therapeutic strategies. We explored several bioactive compounds, such as thymoquinone, allicin, resveratrol, EGCG, piperine, and curcumin, each showing promising anticancer effects targeting multiple cancer hallmarks. Understanding the mechanisms of these compounds can help optimize their use and expand their application in treating various cancer types.

## Figures and Tables

**Table 1:** Bioactive compounds in food.

Bioactive Compound	Food Source	Targeted Hallmarks	Type of Cancer	Concentration/ Dose Used	Experimental Model	References
Thymoquinone (TQ)	*Nigella sativa* (Black seed)	Resisting cell death, enabling replicative immortality, and sustaining proliferative signaling	Hepatocellular carcinoma	20 mg/kg	In vivo	[76]
			Esophageal cancer	5–10 mg/kg	In vivo	[80]
Resveratrol	Grape skin and seeds, peanuts, berries, and tea	Reprogramming cellular metabolism, resisting cell death, sustaining proliferative signaling, and enabling replicative immortality	Human breast cancer	2.5–10 μM	In vitro	[101]
			Colon cancer	12.5–200 μmol/L	In vitro	[102]
Piperine	*Piper nigrum*, *Piper longum*, *Rhododendron faurie*, *Vicoa indica*, *Anethum sowa*, *Fructus piperis longi*, and *Careya arborea*	Enabling replicative immortality, resisting cell death, activating invasion and metastasis, and sustaining proliferative signaling	Colon cancer	75–150 μM	In vitro	[112]
			Ovarian cancer	8 µM	In vitro	[115]
Genistein	Soy products and plants, including alfalfa, barley meal, broccoli, cauliflower, sunflower seeds, caraway, and clover seeds	Enabling replicative immortality, resisting cell death, and activating invasion and metastasis	Triple-negative breast cancer	5–20 µM	In vitro	[122]
			Human colon cancer	25–100 µM	In vitro	[124]
			Hepatocellular carcinoma	10 and 20 µM	In vitro	[125]
Allicin	White garlic (*A. sativum* L.), elephant garlic (*A. ampeloprasum* L.), wild garlic (*A. ursinum* L.), field garlic (*A. vineale* L.), and alpine leek (*A. victorialis* L.)	Sustaining proliferative signaling, resisting cell death, inducing or accessing vasculature, avoiding immune destruction, and activating invasion and metastasis	Human promyelocytic leukemia, and human myelomonocytic leukemia	5 μM	In vitro	[130]
			Thyroid cancer	10 μM	In vitro	[132]
			Cholangiocarcinoma	40 µM	In vitro	[133]
			Lung adenocarcinoma	15 and 20 µM	In vitro	[134]
Epigallocatechin gallate (EGCG)	Green tea, black tea, oolong tea, and in small amounts in various fruits and vegetables	Sustaining proliferative signaling, resisting cell death, and inducing or accessing vasculature	Colon adenocarcinoma	5–40 µg/mL	In vitro	[137]
			Nasopharyngeal carcinoma	40 µM	In vitro	[138]
			Breast cancer	30 µmol/L	In vitro	[139]
			Lung adenocarcinoma	0.5 µM	In vitro	[140]
			Head, neck, and breast carcinomas	30 µg/mL	In vitro	[141]
Curcumin	Rhizome of *Curcuma longa* (turmeric) and other *Curcuma* species	Enabling replicative immortality, resisting cell death, activating invasion and metastasis, and sustaining proliferative signaling	Non-small-cell lung cancer	0.25 and 0.5 µM	In vitro	[145]
Emodin	Root and rhizome of *Rheum palmatum*	Resisting cell death, enabling replicative immortality, sustaining proliferative signaling, and activating invasion and metastasis	Non-small-cell lung cancer	5 µM	In vitro	[152]
Parthenolide (PTL)	Feverfew plant (*Tanacetum parthenium*)	Resisting cell death, sustaining proliferative signaling, and enabling replicative immortality	Renal cell carcinoma	4 and 8 µM	In vitro	[161]
			Lung cancer	1–50 µM	In vitro	[162]
			Cervical cancer	6 µM	In vitro	[163]
Luteolin	Vegetables and fruits such as celery, parsley, broccoli, onion leaves, carrots, peppers, cabbages, and apple skins	Resisting cell death and sustaining proliferative signaling	Human colon cancer	40 µmol/L	In vitro	[165]
			Tamoxifen- resistant breast cancer	10, 20, and 30 μM	In vitro	[166]
			Glioma	30 µM	In vitro	[167]
Quercetin	Fruits, and vegetables, including onions, buckwheat, and broccoli	Resisting cell death, sustaining proliferative signaling, enabling replicative immortality and inducing or accessing vasculature	Breast cancer	10–175 µM	In vitro	[170]
			Leukemia	25, 50 and 100 µM	In vitro	[171]
			Oral squamous carcinoma	50–200 µM	In vitro	[173]
			Nasopharyngeal carcinoma	14.8 and 52.1 µM	In vitro	[174]
Anthocyanins	Fruits and vegetables, including berries, pomegranates, and rice	Resisting cell death, sustaining proliferative signaling, inducing or accessing vasculature, and activating invasion and metastasis	Lung cancer	6.25 and 100 µg/mL	In vitro	[179]
			Colon cancer	100 µmol/L	In vitro	[181]
			Liver cancer	100–200 µg/mL	In vitro	[184]

## Data Availability

The data presented in this study are available on request from the corresponding author (accurately indicate status).
